# From the Brazilian Income Transfer Program to Brazil Assistance: challenges and achievements according to a theory-driven evaluation research on the program

**DOI:** 10.1590/0102-311XEN207922

**Published:** 2023-07-17

**Authors:** Delaine Martins Costa, Rosana Magalhães, Maria Lúcia de Macedo Cardoso

**Affiliations:** 1 Escola Nacional Saúde Pública Sergio Arouca, Fundação Oswaldo Cruz, Rio de Janeiro, Brasil.

**Keywords:** Poverty, Health Status Disparities, Public Policy, Social Programs, COVID-19, Pobreza, Desigualdades em Saúde, Proteção Social, Programas Sociais, COVID-19, Pobreza, Desigualdades en la Salud, Política Pública, Programas Sociales, COVID-19

## Abstract

The recent COVID-19 pandemic has led many countries to adopt emergency cash transfer policies as a way to contain the economic and humanitarian crisis. Such initiatives were developed in a context of exacerbated gender, race, ethnicity and class inequalities resulting from physical and social distancing measures. The article analyzes, by means of an exploratory study and the so-called theory-driven evaluation of the program, the theoretical premises of the Brazilian Income Transfer Program (2003), Brazilian Emergency Assistance (2020) and Brazil Assistance (2021) programs, and their corresponding implementation dynamics. As cash transfer programs are given centrality in the contemporary public agenda, the conclusion is that evaluating their limits and advances - as to theoretical conception and mechanisms triggered in each context - contributes to trace evidence about their effectiveness in addressing long-term inequalities and those inequalities that arise in health emergency contexts.

## Resumo

A recente pandemia de COVID-19 levou muitos países a adotarem políticas emergenciais de transferência de renda como forma de conter a crise econômica e humanitária. Tais iniciativas foram desenvolvidas em um contexto de agudização das desigualdades de gênero, raça, etnia e classe em decorrência de medidas de isolamento físico e social. O artigo analisa, a partir de um estudo exploratório e da chamada avaliação baseada na teoria do programa, as premissas teóricas dos programas Bolsa Família (2003), Auxílio Emergencial (2020) e Auxílio Brasil (2021) e suas respectivas dinâmicas de implementação. Na medida em que os programas de transferência de renda ocupam lugar central na agenda pública contemporânea, conclui-se que a avaliação de limites e avanços, tanto em sua concepção teórica como em relação aos mecanismos disparados em cada contexto, contribui para revelar evidências sobre sua efetividade no enfrentamento das desigualdades duráveis e daquelas que surgem em cenários de emergência sanitária.

## Resumen

La reciente pandemia de COVID-19 llevó a muchos países a adoptar políticas de emergencia de transferencias monetarias como una forma de contener la crisis económica y humanitaria. Dichas iniciativas se desarrollaron en un contexto de profundización de las desigualdades de género, raciales, étnicas y de clase como resultado de las medidas de aislamiento físico y social. El artículo analiza, a partir de un estudio exploratorio y de la llamada evaluación basada en la teoría del programa, los supuestos teóricos de los programas Bolsa Familia (2003), Ayuda de Emergencia (2020) y Ayuda Brasil (2021), y sus respectivas dinámicas de implementación. Dado que los programas de transferencias monetarias ocupan un lugar central en la agenda pública contemporánea, se concluye que la valoración de los límites y avances, tanto en su concepción teórica como en relación con los mecanismos desencadenados en cada contexto, ayuda a revelar evidencias sobre su efectividad para enfrentar las desigualdades duraderas y las que surgen en escenarios de emergencia sanitaria.

## Introduction

The pandemic caused by the SARS-CoV-2 has systemically impacted social and economic relations, with structural effects on public health and the outreach of primary health care services. The multiple impacts of COVID-19 on unemployment rates, worker productivity, and consumption dynamics have exacerbated social inequalities. The reduction of social spending based on fiscal austerity ^1^, science denialism and the expansion of conservative policies ^2^ have made inescapable the debate about the nature of poverty, its repercussions on the control of the epidemic, and the possibilities for overcoming it. The points of contact and the distance between discourses on risk, market failures and human rights with regard to social protection gained prominence and resonance.

Accordingly, income guarantee has been assigned priority on the political agenda. The Brazilian Income Transfer Program (*Bolsa Família*, in Portuguese), created in 2003, was reexamined in relation to other possibilities such as the institution of universal income policies, with or without conditions ^3^ ^,^ ^4^. Some challenges, such as increasing the benefit’s value, expanding coverage, and better monitoring education and health-related conditions, were discussed in a context of political polarization, increasing number of deaths by COVID -19, and economic crisis ^5^ ^,^ ^6^.

The Brazilian Income Transfer Program, regulated by *Law n. 10,836/2004* ^7^ and by subsequent regulations, was characterized as one of the largest and most important conditional cash transfer programs for populations in situation of poverty and extreme poverty ^8^. The benefits provided for in previous programs - School, Food, and LPG gas assistance packages as well as meal voucher - were combined and the Single Registry (*Cadastro Único* - CadÚnico, acronym in Portuguese) was established, which enabled the inclusion of information on Brazilian families in order to guide the formulation and implementation of public policies. At the same time, cash transfer was conditional on social requirements aimed at improving access to health care, education, and social welfare services.

The program was ended in 2021 and, in the same year, the Brazil Assistance program (*Auxílio Brasil*, in Portuguese) was instituted through *Law n. 14,284/2021* ^9^. The Brazilian Income Transfer Program was ended in a socioeconomic context of exacerbation of social inequalities, especially gender, race, ethnicity and class inequalities, as a result of physical and social distancing measures ^10^.

The pandemic increased social vulnerabilities, since the economic recession in Latin America and the Caribbean in the period was the largest since the Second World War, as pointed out by a study by the Economic Commission for Latin America and the Caribbean (ECLAC) ^11^. In the region, it is estimated that the total number of employed people and the labor force participation rate decreased by 9% and 4.8% respectively in 2020. The same study indicates that extreme poverty reached 12.5% and poverty reached 33.7% of the population and affected mainly children, youth and women.

As alternatives to deal with this situation, many countries have strengthened social protection and cash transfer policies. In Brazil, on April 2, 2020, the government, through *Law n. 13,982/2020* ^12^, established the Brazilian Emergency Assistance program (*Auxílio Emergencial*, in Portuguese) in the amount of BRL 600.00 with a validity of three months and focus on individuals living on less than half a minimum wage, unemployed people, informal workers, and mothers in single-parent families. In some cases, such as that of women heads of families, it was possible to receive up to two quotas totaling BRL 1,200.00. However, as of September 2020, through *Provisional Measures n. 1,000/2020* ^13^ and *n. 1,039/2021* ^14^, the benefit amounts were revised and progressively decreased, from BRL 600.00 for three months to BRL 300.00 for four months. In 2021, the benefit was BRL 250.00 for four months.

The Brazilian Emergency Assistance program mitigated the impact of the economic crisis on the most vulnerable families, as well as on the upper classes, through indirect effect and, therefore, contributed positively to mitigate the effects of the pandemic. However, several analysts have warned about the importance of long-term measures to ensure adequate social protection in the country ^15^.

The effects of the Brazilian Emergency Assistance program still need to be investigated. In this study, the aim is to analyze the process of transition from the Brazilian Income Transfer Program to Brazil Assistance program by means of the so-called theory-driven evaluation, which combines a set of authors from the Social Sciences and from the field of Evaluation, on the analytical distinction between the theoretical premises of the programs and the dynamics of implementation.

## Theoretical-methodological approach

Based on authors who dialogue with the evaluation of public programs and policies, socio-anthropological analyses of social inequalities and critical realism, the work presents reflections on the social changes intended in the theory of the Brazilian Income Transfer Program ^16^ ^,^ ^17^ ^,^ ^18^ ^,^ ^19^ ^,^ ^20^, and on the implementation of Brazilian Emergency Assistance and Brazil Assistance programs, and possible effects on poverty reduction.

The program theory-driven approach aims at the analytical distinction between the program’s set of premises and its implementation process ^21^. In this regard, it enables explanatory advances in the understanding of complex and intersectoral interventions, such as conditional cash transfer policies in the areas of health and education. The relation between program theory and implementation theory tends to be a point in common among many authors, in addition to the emphasis on the methodological approach of the social sciences and the focus on the dynamics of cause and effect relations or the so-called “generative causations” ^18^, understood as the social changes intended and achieved by the program. This debate on program theory and its interrelation with the field of Evaluation, as well as analyses on implementation theory and Brazilian Income Transfer Program, can be reviewed in Bodstein ^22^, Costa & Magalhães ^23^ ^,^ ^24^, among others.

In this analytical framework, there is emphasis on social processes and the construction of public agendas ^19^. There is reasonable consensus that social changes are induced by programs, as pointed out by Pawson & Tilley ^18^. The results of a program are seen as contingent and, therefore, are neither fixed nor fully predictable, which implies conducting evaluation research that considers the depth of the stratified social reality (ontological dimension).

For this study, the selected empirical material has different dimensions. The Brazilian Income Transfer Program, established in 2003, was the subject of several research and investigative efforts; therefore, their results could be monitored, compared and evaluated. The Brazilian Emergency Assistance program, of short duration, has several mechanisms aimed at mitigating the effects of the pandemic. In turn, the Brazil Assistance program was established in the late 2021 in a context of fierce electoral clashes and without an explicit formulation on the changes to be achieved and the evaluation strategies to be adopted.

The article is structured into three sessions. The first session presents a succinct analysis of the debate on poverty and social inequality correlated with the proposed theoretical framework. Next, there is an overview of the Brazilian Income Transfer Program considering the theory-driven approach, its implementation and the main results. Finally, there are reflections on health care and social inequalities, considering the post-pandemic challenges. In the conclusions, we highlight the cumulative experience of the analyses of the Brazilian Income Transfer Program and what these lessons warn us about the Brazilian Emergency Assistance and Brazil Assistance programs.

## Poverty and social inequalities: brief approach

Poverty is not considered as a state, but a phenomenon consisting of multiple objective and subjective dimensions, intertwined with the social categories of class, gender, race/ethnicity and generation, which involve different mechanisms, visible and invisible, producing change over time, not necessarily linearly. Within the framework of critical realism, the mechanism is understood as something that can cause effects or make something happen in the world ^25^. It is understood that social categories are constitutive of the social structures that reproduce inequalities.

In general terms, the debate on poverty draws attention to the different factors that affect living conditions. Monetary income, despite presenting difficulties for measurement and establishment of consensus for the definition of “poverty lines”, is seen as a relevant information especially in modern economies. It is also one of the main mechanisms of access to goods and services that can affect living conditions.

According to Himmelfarb ^26^, the “idea of poverty” is a hybrid concept: a cross between aspects of social history and intellectual history. The author presents the relative concept of poverty, highlights the ambiguity of the word “poor” over the centuries and the transformation of “natural” misery into a political issue and a social problem. This path, marked by the denaturalization of poverty, required new positions and multiple perspectives of analysis. Studies on inequalities began to problematize social distances, the different levels of monetary and non-monetary wealth, and to examine the challenges for the advancement of decommodified spheres and population rights. There was a reconfiguration of the debate on the role of Welfare States and democratic societies in overcoming poverty, renewing contemporary interest on the subject, especially after the 1980s ^27^.

According to the analysis of Tilly ^28^, systematic or persistent inequalities define different relational categories, which can be created, transformed and even disappear over time. For the analysis of inequalities, the author proposes relational models of social life, starting with the understanding of “interpersonal transactions” or “social bonds”. Therefore, the author seeks to understand the causal mechanisms underlying categorical inequalities, which operate in the domain of collective experience and social interaction.

In Brazil, the theoretical perspectives that approach inequalities only from a class perspective are insufficient as an explanatory axis ^29^ ^,^ ^30^ ^,^ ^31^ ^,^ ^32^. Sociological studies have shown that increased income and consumption do not represent social inclusion, and that universal and focal policies complement each other ^33^. On the other hand, studies on social stratification and mobility indicate how the labor market formation has reproduced and reinforced ethnic-racial and gender inequalities ^34^.

As Brito ^35^ points out, since the mid-1990s a new trend has arisen in Latin America that can be defined as the provision of monetary income to poor families associated with investments in human capital, such as ensuring the school attendance of children and youth and access to health care services. Such public strategies, referred to as conditional cash transfers, sought to mitigate situations of deprivation and structural poverty, improving access to services capable of breaking the intergenerational circuits of misery.

However, since the 1990s the universalization of cash transfer benefits versus their focalization - that is, the creation of criteria to reach specific groups of the population living in poverty - has constituted one of the main dilemmas of social protection policies ^36^. Advocates of focalization argue as to the efficiency and equity in the use of scarce resources. However, the focalization mechanism - instead of the perspective of universalization - is questioned since administrative costs tend to be high and compromise spending on expanded benefits, in addition to undermining the policy’s fiscal sustainability through fragmentation or duplication of benefits for the target population.

Poverty, social inequalities and vulnerabilities pose conceptual and methodological challenges for the formulation and implementation of public policies. Cash transfer policies can be understood as a response to this challenge, within the framework of the debate on social protection. Direct transfers enable beneficiaries to choose to use the resource in any way that suits them. Although arguments about the “improper” use of the resource are highlighted in different studies, in general, it is recognized that the initiative fosters productive activities and stimulates the market ^36^. However, studies indicate that the impact of cash transfer on poverty reduction is small, due to the low value of resources and the often short period of the benefit. For longer-lasting impacts, such studies point to the need to increase the value of the benefit and ensure its continuity ^37^.

In Brazil, the *Federal Constitution* (1988) ensured a social protection system (social security, social aid, and health care) for all citizens, guaranteeing public, universal, and free access. However, as pointed out by Lavinas ^38^, social spending takes the form of cash transfers and the provision of decommodified services remains at a minority level and below what is established by law. Therefore, the adopted social model neglects mechanisms aimed at reducing inequalities. Still, conditional cash transfer programs innovate in terms of tackling poverty.

## The Brazilian Income Transfer Program “theory”

From the perspective of program theory-driven evaluation, it is necessary to describe the objectives, activities and resources provided for, and determine the mechanisms through which the intervention design operates or is intended to operate. Comparing programs is relevant, as it enables understanding the consistency of the theoretical premises that support the different initiatives and also the practices adopted in each context. Differently from the expectation that the program's logical framework provides a clear representation of the causal links between activities, resources and results, the theory-driven evaluation explores ambivalences, controversies and inconsistencies both in the program’s normative design and implementation process. The local context is seen as a constitutive analytical dimension of the programs, as it sheds light on the dynamics involving cooperation and conflict of interest, which hinder or further the achievement of the objectives.

If we take into consideration the Brazilian Income Transfer Program theory, it is possible to perceive some contradictions and tensions with regard to the intended effects and the prospect of reducing inequalities. We know that the Brazilian Income Transfer Program’s explanatory statement in *Law n. 10,836/2004* provides central aspects: the target public are poor and extremely poor families, according to income criteria; expansion of access to universal policies (education, health care, and food); combat against hunger and poverty by meeting basic needs and inducing access to social rights; establishment of conditions understood as mechanisms and social requirements ^8^.

However, based on the literature on program theory, it is worth asking ^39^: how is the program expected to produce changes and what changes are intended? Two important directives can be observed. One was the adoption of mechanisms geared toward guaranteeing complementarity and comprehensiveness (whose lack can be considered as grounds for the criticism directed at previous programs), in order to break the sectoral and departmental logic that sprays resources, overlaps actions, leads to institutional conflict, and causes fragmentation. The other directive referred to the concept of poverty seen as a complex and multidimensional phenomenon that cannot be combated in a lasting manner only with cash transfers. Therefore, the success of the Brazilian Income Transfer Program would depend on the combination of emergency actions with structural policies, in addition to the combination of efforts of state governments and organized civil society. In summary, the program theory indicates the coordination between emergency actions (cash transfers) and structural policies leading to the reduction of poverty and hunger. The intended social change refers to the inclusion of beneficiary families in health care, education and social welfare services and policies.

The Brazilian Income Transfer Program makes use of vulnerability factors and correlates them to the families’ social requirements, seen as “concrete elements for their socioeconomic emancipation”. In addition to the conditions, it emphasizes the complementary programs that include actions in the areas of training and microcredit to be implemented in coordination with state governments.

The main goals of change are the fight against hunger and poverty, considered in its multidimensionality. Hunger was seen not only as a consequence of poverty, but as a result of inadequate and/or discoordinated public policies in the areas of health care, education and social welfare. In turn, making the degree of food insecurity measurable and visible requires a set of intersectoral actions in order to provide food, including in schools and daycare centers, and monitor child development. On the other hand, the cash transfer itself does not mean adherence to the intended changes, which demands requirements; families were induced to comply with a monitoring schedule in the preestablished but not necessarily preexisting service network. This adherence also involved managers and professionals working in an intersectoral manner. The scope of the intended changes involved families, and women (and mothers) were recognized as the main responsible for the care and the task of converting income into well-being.

The choice of having the financial benefit be registered in the name of women follows an international trend, either by recognizing the increasing number of women heads of families, especially among the poorest; or by the sexual division of labor that entails a double shift for women; or because women, in caring for children and the household unit, face more difficulties to enter the labor market. Other factors can also be listed, such as domestic violence and gender discrimination, which contribute to making women more vulnerable. Thus, making them holders of the benefit can be seen, in the logic of program theory, as a more effective mechanism for state control over compliance with conditions. When they become holders, to some extent, they become responsible for the financial management of resources; however, on the other hand, they also become the main person accountable for compliance with requirements, which reinforces gender inequalities.

The conditional cash transfer in the scope of the Brazilian Income Transfer Program imposed changes at multiple levels, since it involved the fulfillment of conditions in the health and education areas and, it can be affirmed, required the coordination of actions with the other components of the program and the development of an intersectoral network. At the origin of the Brazilian Income Transfer Program, the objective of focalization resulted in a better inclusion of poor and extremely poor families and, therefore, in the provision of more accurate information in the CadÚnico registry system. For better integration of the social protection system, the program provided that the implementation should be conducted in a decentralized and intersectoral manner. A key aspect was the development, in 2004, of the Brazilian National Social Assistance Policy (PNAS) and, in 2005, of the Brazilian Basic Operational Standard of the Unified Social Assistance System (NOB/SUAS). Subsequently, 2008 saw the institution of the Conditionality System ^40^.

Some weaknesses persisted. The complementary programs associated with the Brazilian Income Transfer Program program and aimed at the development of labor capacities lacked institutional coordination and political priority in their implementation ^38^. In order to overcome these weaknesses, there was the development of the Brazil Without Misery Plan (2011) and the institution of the Extraordinary Secretariat for the Overcoming of Extreme Poverty (SESEP), responsible for coordination activities.

**Figure 1:**
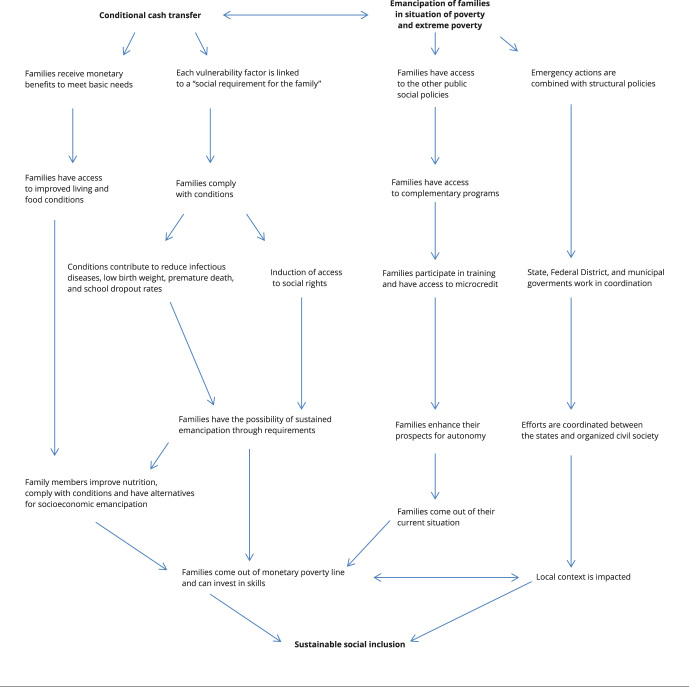
Theory of the Brazilian Income Transfer Program.

Figure 1 presents a summary of the Brazilian Income Transfer Program theory ^41^.

Despite socioeconomic inequalities between regions, communities and population groups, since 1990 the Brazilian health system has incrementally achieved greater coverage and outreach in health care ^42^. The Family Health Strategy (FHS) increased the availability, access and use of health services, while producing better health indicators. However, inequalities have increased in terms of barriers to access, especially among individuals with lower income and education levels, and without formal employment ^43^.

The association between the FHS and Brazilian Income Transfer Program enabled new devices to reach the poorest; however, difficulties still persisted, either in terms of the scope of cash transfers, access to basic health services, or both. The health condition of populations can be a good measure to understand inequalities in a country and, in this regard, in Brazil, social advances have not been made in an equitable manner, which is why it is relevant to observe how the Brazilian Income Transfer Program and other social programs contribute not only to the improvement of health, but also to the reduction of inequalities ^44^.

Despite the evidence on the positive effects of the FHS, some challenges remain. The main explanatory reasons include the low number of professionals, municipal budget constraints, and precarious infrastructure. However, in a study that analyzed its expansion and coverage in Brazilian municipalities, between 1998 and 2012, the positive impacts suggest the improvement of local health systems ^45^. By making use of conditional cash transfers as a proxy for measuring poverty in municipalities, the survey found an increase from 22% (2004) to 36% (2012) in the Brazilian Income Transfer Program coverage. Despite the heterogeneity and different coverage patterns among municipalities, the results of the research indicate that municipalities with high Brazilian Income Transfer Program coverage demand more social programs. With the FHS, other benefits are made available, but the poorest municipalities often have only public primary health care.

Access to health is considered one of the main mechanisms for breaking the intergenerational cycle of poverty, either because malnutrition has concrete and severe consequences for child development, or because the absence of primary health care can produce irreducible consequences throughout the life cycle. These and other aspects reinforce the importance and potentiating effects of the coordination between the Brazilian Income Transfer Program and the FHS since, in many municipalities, the conditional cash transfer policy finds, in the FHS, the mechanisms for compliance with the required health conditions and for improvement of the health conditions of the beneficiary population.

Attention to food insecurity rates, vaccination coverage, basic health care for pregnant women, nursing mothers, children and adolescents are practices that already exist in the health policy, but that have become the object of monitoring in conjunction with the poverty reduction policy ^46^ ^,^ ^47^.

According to a recent study, based on the traditional *Brazilian National Household Sample Survey* (PNAD), from 2001 to 2015, and Continuous PNAD, from 2016 and 2017 ^48^, 70% of Brazilian Income Transfer Program’s resources reached 20% of the poorest. Despite the small budget (0.5% of gross domestic product - GDP) and the limited participation in the total income of PNAD families (0.7%), the same authors say that the program had a relevant impact: its transfers reduced poverty by 15% and extreme poverty by 25%.

With regard to the Brazilian Income Transfer Program theory, conditional cash transfer was consistent in a context in which public health, through the expansion of FHS coverage, enhanced the intended effects, although it is questionable to treat a right as a social requirement. On the other hand, the expansion of programs aimed at the autonomy of families seems to have been the great problem of the Brazilian Income Transfer Program: if the low value of the benefit associated with other policies contributes to reduce poverty and extreme poverty, the same does not occur for these families to be able to access the labor market and, consequently, guarantee the sustainability of efforts to overcome social vulnerability.

Some challenges remain related to protection policies: the financialization of social policies and the consequent underfunding of health policies ^49^; the weak redistributive effect due to the low value of the benefit ^47^; and the accelerated rise in poverty combined with the disruption of the labor market. A key factor refers to the particularities of the dynamics of income generation among poor and extremely poor families, that is, volatile income and unstable participation in the formal and informal labor market ^47^ ^,^ ^49^ ^,^ ^50^.

## Brazilian Emergency Assistance and Brazil Assistance: changes and continuities in the context of increased inequalities

Cash transfer measures can be seen as an effective epidemiological strategy, considering the context of the COVID-19 pandemic and its multiple and systemic effects ^15^. According to the different regulations published in the Brazilian Emergency Assistance: people eligible to receive the benefit included those aged over 18 years, with income of up to half a minimum wage per capita or a family income of up to three minimum wage, limited to two quotas per family, and women who provided single-parent families could receive two quotas of the aid. Except for the Brazilian Income Transfer Program, the individual could not have social security or welfare benefit ^51^. The Brazilian Center of Analysis and Planning (CEBRAP) ^34^ analyses reported that 10.6 million Brazilians had no income, depending only on the emergency aid, and that these were 5% of the Brazilian population and, among them, 67% were black.

When defining certain social groups as eligible, it becomes relevant to consider the heterogeneity of the inclusion and of the conditions and life of workers in the labor market. Precarious conditions of housing, sanitation, infrastructure and transport have direct implications on the physical distancing that, associated with the health protocols, affected the measures to be followed during the different times of the pandemic.

The Brazilian Income Transfer Program theory indicates that emergency actions must be combined with structural policies, based on the coordination of state governments and civil society, in addition to conditions, which did not occur in the Brazilian Emergency Assistance benefit. The health policy during the health emergency to combat COVID-19 was not directly linked to the benefit. For example, the lack of free virus detection tests and specific guidelines within the scope of the FHS may have contributed to increase the difficulty of inclusion and permanence of workers in the labor market, which was aggravated by the delay in immunization and even by it not being considered a requirement to receive the aid.

On the other hand, studies indicate that the articulation with civil society organizations allowed mitigating some effects arising from the low intersectoral coordination. If the impact of the emergency aid is not directly related to health, but to the economy, consumption and employment ^51^, it is necessary to deepen the analysis of its effects considering the dimension of race, the double workday that affect women, the implications of physical and social distancing on mental health, and the care to be provided given the post COVID-19 effects among those who have been infected or who have lost family members, many of them responsible for economic livelihood.

It is also worth investigating the epidemiological consequences of COVID-19 on the population, in an intersectional approach, in addition to the clinical trials that specifically address this subject and the capacity of the primary health care network to absorb new demands stemming from the long-term effects of the disease. Since 2016, a decline in immunization rates and coverage levels against measles and other diseases has already been observed ^52^. Despite the recognition of the disruptive effect of COVID-19 on health and services, fiscal austerity measures have also caused negative impacts on Brazilian Unified National Health System (SUS). The underfunding of a universal health care policy causes, in the short term, an increase in social inequalities in the population as a whole.

The context in which the transition from Brazilian Income Transfer Program to Brazilian Emergency Assistance and to Brazil Assistance occurred still requires in-depth studies and research. In the program theory-driven approach, the local panorama is a key aspect for understanding the context-mechanism-result configurations ^18^ ^,^ ^53^. The context in which the Brazil Assistance was implemented is central to reflect on the social changes intended and achieved. Context - understood as an irreducible set of factors that influence when and how an intervention is implemented and the mechanisms triggered in the local reality ^18^ - deserves consistent analyses. Recognizing social contexts as dynamic, contingent and non-linear is a central aspect to understanding the outreach and limits of actions and programs ^54^.

The changes intended by the Brazil Assistance are not limited to social welfare policies or projects to reduce social inequalities, but concern broader conceptions about the dynamics of social processes in which some elements (symbols, ideologies, rituals and narratives) have gained prominence amid political and partisan disputes in the recent Brazilian conjuncture.

The use of nationalist symbols such as the Brazilian flag and the permanent reference to the idea of fatherland express a tendency to homogenize and naturalize the process of sharing meanings, which can obscure the constitutive multiplicity of society. At the same time, the appreciation of the notion of aid as something more fluid, transitory and distanced from the language of social rights of citizenship tends to reveal a challenging environment for the construction of equitable public policies. In addition, there is a symbolic break from the name of the Brazilian Income Transfer Program and, in contrast, an identification with the Brazilian Emergency Assistance, conveying the notion of continuity with the cash transfer implemented during the pandemic, reaffirming the conception that it is an occasional benefit and dissociated from a long-term prospect.

The Brazil Assistance is also marked by the absence of strategies for dialogue with organized civil society. Maria Emília Pacheco, in an interview with the Humanitas Unisinos Institute ^55^, points out the main criticisms concerning the program and highlights the note of the Brazilian Bar Association (on the change of law on the bonds issued to finance court sentences against the government, source of the program’s resources), and the open letter for the rejection of *Provisional Measure n. 161*, signed by a few hundred civil society entities. The dismantling of the country’s social protection system, the denial of the experiences accumulated by the Brazilian Income Transfer Program and by the Brazilian Food Acquisition Program are noted, in addition to the neoliberal vision that guides current policies. Lessons learned from previous cash transfer programs in the country and in the world are not valued and evaluation strategies are not incorporated, hindering future analyses of the evidence of effectiveness of actions.

In the program theory, the Brazil Assistance indicates the integration of public policies (social welfare, health care, education, employment and income) as an intended change, as had occurred in the Brazilian Income Transfer Program. The criteria for inclusion in the Brazil Assistance are similar to those of the Brazilian Income Transfer Program; however, emphasis is given to the “simplification of the basket of benefits” for the emancipation of families, achievement of autonomy, and overcoming of social vulnerability. These aspects were strongly present in the theory of the Brazilian Income Transfer Program and constituted the main challenges of the implementation process. The Brazil Assistance theory continues to prioritize pregnant and nursing women, as in the Brazilian Income Transfer Program, but the reasoning is different: instead of recognizing the increased number of in single-parent families and the difficulties experienced by women heads of families, the benefit can be seen as a “compensation” for this condition. The target public, as in the Brazilian Income Transfer Program, also consists of children and adolescents, and it is proposed that the financial benefit called Brazilian Child Citizen Assistance (*Auxílio Criança Cidadã*, in Portuguese) can be used to expand the “provision of child care in daycare centers”, in an induction of its use for payment of private daycare centers. The low level of access to basic education and public daycare centers is not questioned, which limits productive inclusion as well as its coordination with education policies and its expansion, since it is known that women with children (especially in early childhood) face greater difficulty to enter the labor market. Fostering the “scientific and technological performance of excellence” of children, adolescents and young people is also a measure intended by the Brazil Assistance through specific benefits. However, the program theory is not clear as to how this change will be achieved. Families entitled to Brazilian Child Citizen Assistance are those in situation of poverty (monthly family income per capita between BRL 105.01 and BRL 210.00) and extreme poverty (monthly family income per capita of up to BRL 105.00) and in “rule of emancipation” ^56^. As stated in the Brazil Assistance, there is a “root basket” composed of a set of benefits, described in Box 1, and which can be accumulated.

**Box 1:** Summary of the components of the “root basket” and other benefits of the Brazil Assistance (2022).

NAME	AMOUNT	WHO IS TO RECEIVE IT
Early Childhood Benefit (BPI)	BRL 130.00	Families with children aged up to 36 months
Family Composition Benefit (BCF)	BRL 65.00 per person	Families with: (a) pregnant women; (b) nursing mothers *; and/or (c) people aged 3 to 21 years **
Benefit for Overcoming Extreme Poverty (BSP)	Amount calculated so the per capita family income exceeds the amount of the extreme poverty line, fixed at BRL 105.00 per month per person	Per capita family income exceeds the amount of the extreme poverty line
Compensatory Transition Benefit (BComp)	The total amount of the Brazilian Income Transfer Program benefits received by the family in the month prior to the end of the program will be considered	n/a
Junior Scientific Initiation Scholarship	I - 12 monthly installments of BRL 100.00 for the student; and II - single installment of BRL 1,000.00 per family	Students, members of families beneficiaries of Brazil Assistance, who have excelled in academic and scientific competitions of national scope. The amounts are the same as the Brazilian School Sport Assistance
Brazilian School Sport Assistance	I - 12 monthly installments of BRL 100.00 for the student; and II - single installment of BRL 1,000.00 per family	Financial aid granted to students aged 12 to 17 years, members of families beneficiaries of the Brazil Assistance program, who have excelled in the Brazilian School Games
Rural Productive Inclusion Assistance	Benefit paid in monthly installments of BRL 200.00	Families served by the Brazil Assistance program that include family farmers. Proof of classification as a family farmer will occur by the Declaration of Eligibility for the National Program for Strengthening Family Agriculture. Payment of more than one aid per person and per family is prohibited
Urban Productive Inclusion Assistance	Information not available	

n/a: not applicable.

Source: information systematized by the authors based on the Brazilain Ministry of Citizenship data ^56^ ^,^ ^60^.

* For pregnant women, the benefit will be ended after the generation of the 9th installment. In order for the BCF to be granted to nursing mothers, the family must update the information on the birth of the new child in the Single Registry before the child completes 7 months of life. Payment of the benefit ends after the sixth installment;

** The family will only receive this benefit relative to its members aged between 18 and 21 years if they are enrolled in or have completed basic education.

Despite a reasonable consensus that cash transfer, improved consumption and access to services are crucial to overcoming poverty, the ability to address vulnerabilities is extremely more complex and requires reviewing the focus and scope of different public initiatives. The daily life routine of poor families and individuals shows a structural and long-lasting adaptation to the most distinct forms of material and symbolic deprivation. Cash transfer, in this regard, should be analyzed as a partial response to the challenges of social vulnerability. The priority issue is then how to design policies and programs that support different vulnerable groups and reduce risks and, at the same time, rethink how such contexts of misery arise and remain for decades. Chronic poverty can involve individuals who are sick, have different disabilities, are discriminated against due to race/color or gender, are subject to domestic violence, are migrants and have been unemployed for a long time. A broader range of social protection strategies implies recognizing multiple situations of vulnerability not necessarily mitigated by cash transfers. According to Barros, in an interview with the newspaper *O Globo* ^57^, Brazilian Emergency Assistance and Brazil Assistance have an information deficit. According to the researcher and one of the formulators of the Brazilian Income Transfer Program, it is crucial to recognize the scrapping of CadÚnico and the distancing of the Social Assistance Reference Centers (CRAs) from the most vulnerable groups. As a result, the possibility of understanding the multidimensionality of poverty and the different needs of poor families was lost.

As previously analyzed, the country needs cash transfer programs and, even more, the prospect of associating the benefit with social requirements that foster increased school education, productive activity, and access to health services. Collective actions geared toward workers’ rights, expansion of microcredit, massive investments in education and health, and strategies against discrimination can permanently and comprehensively transform the situation of misery ^58^.

Evaluation researches and especially those based on program theory can occupy a central place in the development of conditional cash transfer policies. In this regard, it is observed that, in addition to the spraying of resources without clear directives for the policy, no resource is provided for evaluating the effectiveness of the actions. Having such interventions as an object of evaluation requires an investigative question about the nature (or ontology) of the programs, which requires conceptualizations and knowledge about the design and execution of actions or interventions to be implemented in the specific social contexts. The Brazilian Income Transfer Program was the subject of different evaluation studies throughout 18 years. Successes and errors were debated on the basis of evidence. The Brazil Assistance program - by not providing for the evaluation of its achievements and limits - fails to contribute with lessons about the issue of combating poverty in the country.

## Conclusions

Conditional cash transfer programs are public strategies introduced in social contexts. These are complex initiatives that are not limited to a set of isolated components or practices. In addition to establishing associations between the intervention and the effective results, the theory-driven evaluation of programs seeks to leverage the learning process, the formulation of new hypotheses, and the production of knowledge about the complex nature of public interventions. Econometric parameters and those based on the perspective of linear causation do not contribute to the understanding of possible evidence of effectiveness of multilevel actions. Comparing the theories of the programs and the corresponding implementation processes questions the rationalist view of public policies and enables analyzing the consistency of the normative design and the challenges of the local context in which mechanisms are triggered, barriers are faced, and different power arrangements emerge. In turn, the evaluation based on the experimental model produces the description of the results, but does not clarify how the programs operate.

The theory-driven evaluation research ^18^ proposes to answer the following questions: what are the mechanisms for change triggered by the program? And how do these mechanisms neutralize or enhance social processes that exist in the local context? Accordingly, it is necessary to recognize that the results of public programs and policies occur in open systems, that is, there is a redesign of the program’s regulations during implementation and the relation between mechanisms and effects is contingent.

With regard to conditional cash transfer policies, the strategies of public investment, renewal of collective facilities, and improvement of access to education, health care and social welfare should be associated with attentive consideration of the biographical trajectories of the impoverished individuals. The construction of more robust criteria for the distribution of goods and services taking into account the diversity of the individuals’ initial conditions tends to be a guarantee of greater equity ^59^.

At the same time, the introduction of social requirements associated with a monetary benefit can break the false opposition between universalizing programs and programs focused on contexts of extreme poverty. The Brazilian Income Transfer Program contributed to advances in the coverage of the FHS and to the intersectoriality and decentralization of public interventions against poverty. The Brazil Assistance program theory reflects a view of poverty pervaded by the prospect of electoral gains by neglecting previous institutional lessons and not providing solid guarantees of sustainability.

According to the literature on program theory and authors that examine social inequalities in health care, the Brazilian Income Transfer Program contributed to reduce poverty and extreme poverty, as well as enabled, through different mechanisms, a greater outreach of social protection policies. Therefore, by relying on a successful program theory, tested in evaluation studies, conditional cash transfer remains a generative mechanism capable of producing changes.

Investigating the Brazil Assistance program, through the program theory approach, shows the weaknesses of its conception and implementation, and its limitations in facing enduring inequalities and those that emerge from a new context of urgency, which may become permanent. Similarly, proposals currently under discussion and development, aimed at a universal basic income, could also benefit from the analyses on the Brazilian Income Transfer Program.

It is worth emphasizing the importance of a research agenda on the effects of the Brazilian Emergency Assistance and Brazil Assistance programs on the coordination with other social protection programs and, in particular, the FHS. The investigation and analysis of the processes for implementation of actions enable examining the points of contact and distance between discourses and strategies that seek to associate the debate on risks, vulnerabilities and human rights with regard to social protection.

The article was finished and reviewed in the transition of the Federal Government, with the election of Luiz Inácio Lula da Silva as president of Brazil from 2023 to 2026. The debate on guaranteeing the budget for the cash transfer program had major prominence in the period, reaffirming the importance of the topic in the public agenda. Certainly, there will be significant changes in the execution of social protection and, especially, conditional cash transfer programs. We hope that evaluation will occupy a central place in this agenda.
